# Nutrition

**Published:** 1875-11

**Authors:** A. J. Turner


### NUTRITION.

BY A. J. TURNER.

*Read before the Indiana State Dental Society.*

In the study of nutrition there is so much involved that in a paper of the dimensions which we propose to give to this, it would be impossible to present all the interesting phases which offer themselves and which are so closely allied to our subject that they may be considered part of it.

Nutrition: from *nutrio*, to nourish, begins with the food we eat. It is a lamentable fact, which all observers recognize, that too little attention is paid to this beginning of the elements of life. We do not forget that the woman of to-day has changed in many respects, for the progenitors of our race. In no country in the world are women so beautiful as in our own broad land. As the girl develops into womanhood, we begin to realize the beauty of Milton's verse put into the mouth of Adam.

"Oh fairest of creation, last and best,
Of all Gods works, creature in whom excelled,
Whatever can to sight or thought be formed,
Holy, divine, good, amiable, sweet?"

This lovely American woman, with gentle refinement, graceful figure, vivacious, entertaining, charming, develops into a fragile being, to be in a few short years changed into one of premature decay. We shall not stop here to show how the peculiarities of climate, sudden changes in temperature and high pressure mode of living may enter into the causes of this premature decay. Neither have we the time nor inclination to compare the different conditions among the races of earth, in which we find this simple question exemplified. I have just said simple, but it is being so much overlooked, that its very profundity and abstruseness is lost in this very simplicity. The Scotchman has come to regard his oat meal and porridge as much a matter of fact of every-day-life as the Irishman does his potatoes. The proverbial roast beef and plumb pudding of the Englishman, marks his rounded outline and florid complexion, while the savant of the culinary art, the Frenchman, regards no dinner complete without his soup, no matter of what made, even regarding horse hide or old boots as a better base than no base at all, so that he be furnished with his dish of soup. The Italian prefers his maccaroni to the Irishman's potatoes and the American, poor fledgling of humanity on nutrition, forgetting everything but palate and passions, voraciously devours his soda biscuit and mince pies.

Now we lay down the proposition that to understand nutrition, we must remember that food should contain two elements, one to nourish and the other to give heat. And just so much as we may overlook these qualities shall we suffer in the formation of the ultimate tissue and bone. The infant of every race and clime begins with its mother's milk, and simple though it be, it subserves all the necessities of this important and formative period of life. Simon's analysis of mother's milk, makes it consist of water 883.6; butter, 25.3; casein, 34.3; milk, sugar and extractive matter, 48.2; fixed salts, 2.3 equal, 1,000 parts. Here we find all the requisites for the nutriment of the child. We can understand then, how anything which interferes with the formation of healthy milk, will in like proportions, affect the formation of tissue in the infant. For purposes which will disclose themselves as we progress let us now give Mr. Haidlen's analysis of cow's milk: Water, 873; butter, 30; casein, 48.20; milk sugar, 43.90; phosphate of lime, 2.31; phosphate of magnesia, .42; phosphate of iron, 07; chloride of potassium, 1.44; chloride of sodium, .24; soda in connection with casein, 42; equal, 1000.

Let us now return to our proposition that to understand nutrition, we must use food containing the two elements, one to nourish, and the other to give heat. And for the sake of explaining, we will divide the two into, first, albuminous or nutrient; and saccharine and oleaginous, or calorificent, heat giving. According to Dr. James Paul, we find in a chemical analysis the following result:

| | ALBUMINOUS. | | OLEAGINOUS. |
|---|---|---|---|
| | Eggs. | Wheat. | Mutton Fat. |
| Carbon | 55.000 | 55.01 | 78.996 |
| Hydrogen | 7.073 | 7.23 | 11.700 |
| Nitrogen | 15.920 | 15.92 | |

| | ALBUMINOUS. | | OLEAGINOUS, |
|---|---|---|---|
| | Eggs. | Wheat. | Mutton Fat. |
| Oxygen, Sulphur, Phosphorus | 22.007 | 21.84 | 9.304 |

SACCHARINE.

| | Starch. | Arrow Root | Sugar from Starch. | Sugar of Milk. | Cane Sugar. |
|---|---|---|---|---|---|
| Carbon | | 44.40 | 37.29 | 40.00 | 42.301 |
| Hydrogen | | 6.18 | 6.84 | 6.61 | 6.384 |
| Oxygen | | 49.42 | 55.87 | 52.93 | 51.315 |

We see immediately that the peculiarity possessed by the albuminous or nutrient over the oleaginous and saccharine, or heat giving differs in that the albuminous contains nitrogen and that while the oleaginous and saccharine contain oxygen the albuminous contains oxygen in combination with sulphur and phosphorus. We are thus led to infer that by the processes of nature these peculiarities bring us to the point that they possess that which builds up or strengthens the body, while the oleaginous and saccharine furnishes the wood and coal to keep the body at a proper temperature for the ordinary processes to go on. Now we do not over look the fact that climate and habits of life may vary this in its application. Thus the natives of a tropical clime would require more of the albuminous while those of the poles should receive more of the oleaginous. 1,000 parts of healthy blood according to M. Lecamis' analysis gives us the following proportions;

| | | |
|---|---|---|
| Water | 780.15 | 785.58 |
| Fibrin | 2.10 | 3.57 |
| Albumen | 65.09 | 69.41 |
| Coloring matter | 133.00 | 119.63 |
| Crystallizable Fat | 2.43 | 4.30 |
| Fluid Fat | 1.31 | 2.27 |
| Extractive Matter Uncertain | 1.79 | 1.92 |
| Albumen in Combination with Soda | 1.26 | 2.01 |
| Chlorides of Soda and Potassium Carbonates, Phosphate and Sulphates of Potash and Soda | 8.37 | 7.30 |
| Carbonates of Lime and Magnesia Phosphate of Lime and Magnesia and Iron, Peroxide of Iron | 2.10 | 1.42 |
| Loss | 2.40 | 2.50 |

The vast proportions which water maintains in blood is for some wise purpose. Take it away and we have less than one-third of the whole amount left. Then if we add to the water 780 and the coloring matter 133 parts, we have more than 900 parts in these two constituents, leaving less than one-tenth of the whole amount of blood to be distributed among the organic and earthy matter. What a plea we find here for water, a demand which we must constantly supply to give the blood its healthy proportions. No temperance lecturer pleads more eloquently for water than does the blood. No amount of cajoling or bribery will keep it from constantly uttering its demands for water, and no other fluid so well supplies its place. Wines and liquors only stimulate to a degree which makes the call louder and more fierce when they have relaxed their stimulating influence and no fluid having taste or smell, can ever supplant it. How could the red globules or corpuscles float in anything but water, and how could they permeate the infinitesimally small capillary tubes, which they can now enter, without this vessel which floats them and which carries them to organs which must die without their constant food. Sooner let us give up our soda biscuit and mince pies, our mansard roofs and four in hand, than part with the pure limpid fluid which is so lavishly and so freely and without price furnished us by an all wise Dispenser of Gifts.

We have referred to mother's milk. We stated that in it we find everything necessary to the nourishment of the child, of every race and clime and condition. In this question of nutrition we can easily see that if the infant finds in this one article, all it needs, could not man, though more mature, satisfy himself with a simple diet which contains all the constituents that he needs for the constantly recurring demands made by his nature?

Remembering then that the blood must have the nutrient and the heat giving properties, we find that the food which is most demanded is the simplest. Under the nutrient, we should recommend the lean of meats, such as beef, bird or fish and potatoes, beans, eggs, milk and wheat. The meat to be easily digested, should be cooked to a rare. The eggs soft boiled, barely curdling the albumen or white. The milk never skimmed as thereby we loose a large part of the casein. The potatoes cooked with the skin on until soft to the little core in the center, which should remain hard. And lastly the wheat should be made into a flour with the kernel on. According to the Patent Office Report, 1847, page 116 the whole grain is more nutritious by half, than the fine flour which our millers grind and bolt. The same report shows the difference in weight of a barrel of flour without the bran and when only the outer coating of the wheat is taken off. It says: The weight of the bran or outer coating, would, in the common superfine flour, constitute the offal weighing about $5\frac{1}{4}$ pounds to the barrel of flour, while the ordinary weight of offal is 65 to 70 pounds to each barrel of flour, showing a gain of $59\frac{3}{4}$ to 65 pounds of wheat in every barrel thus showing an immense loss in the earthy constituents lost in the offal of the bran.

Still further, chemical analysis of the incisors of man shows:

| | Cementum. | Dentine. | Enamel. |
|---|---|---|---|
| Organic Matter | 29.27 | 28.70 | 3.59 |
| Earthy Matter | 70.73 | 71.30 | 96.41 |

Here we see the loss sustained in using bolted flour when the enamel, that hardest of all bone, has 96.41 parts of 100 of earthy matter.

If we look to the analysis of bone we find the following, viz:

Organic Matter 32.56. Earthy Matter 67.44.

Making in bone, the largest portion consist of earthy matter. Instructive as this may be, it must be put into practice to result in the good which it could do. Just how important this question of food is, no one knows better than the physiologist, and it would seem that just in proportion as its importance increases the races ignore it. Here we may discover one of the sources so prolific and fruitful in producing decayed teeth. The teeth like the muscle, must receive their sustenance from the blood. If the blood be deficient in that which produces earthy matter in the place of healthy crusta petrosa, dentine and enamel, we have only a semblance of it, which, before they have fairly erupted, show signs of decay, in deep fissure cavities and milky and chalky fabrics as easily cut away with a sharp instrument as the surgeon's lance opens an abscess or sections a tumor.

The heat giving principle we find in the usual food, in milk, wheaten bread, potatoes, arrow root, corn and in most vegetable matter and in sugar, anything that abounds in carbon.

We part reluctantly with this part of our subject, because in it we believe we have found the germ which will disclose to us not only the reasons but the remedy, in many instances, for many of the diseases and conditions which have baffled the skill of physicians. It is in vain that we are told that these deficiencies are supplied by the pharmacy. Drugs are not so palatable as food and the latter if properly and in good time applied, might in many instances dispense with doctor's bills and pills.

We should like to stop just here but for the fact that we have said nothing of the processes of digestion, circulation, respiration nor yet of gestation, all of which are correllatives to our subject, but which we must for the present pass by, to devote the remainder of this paper to the process of assimilation. And in considering it we wish to be as practical as its harmony will admit.

First then we assume that assimilation begins when any part of the blood arrives at that condition which enables the tissue to appropriate it. Just how this is done it is not our purpose to enquire.

When the minutest miscroscopical investigation fails to disclose the how, such a question is beyond our present range.

We merely recognize the fact that the blood arriving at a certain condition is metamorphosed by affinities into muscle, bone and enamel. With the exception of the latter disintegration results when its offices have been fulfilled and new pabulum arrives to supply the waste occasioned by this disintegration. Just what this condition is, is best explained when we understand the process of transformation or metamorphosis going on in the organs when the blood is brought into contact with them. There is this peculiarity which we discover in starting upon such a journey with the blood in its circuit through the body. We find in it nothing resembling the organs themselves nor the tissues, bone or enamel. Yet here we see like becoming unlike, and unlike like. A conjugal relationship, if you please, which is ready to take on the solemn and responsible duties of existence so soon as the ministerial *ipse dixit* has pronounced the union of two, one. This ministerial office is performed by the cells of each organ and the metamorphosis occurs when the elements in the blood come in contact with these cells having the appropriating power. This power is limited and is very choice of its affinities, so much so indeed that the cell generally finds but one element in the blood for which it seems to care at all. There may be many other elements there awaiting an invitation to come in but they wait in vain, until they have been carried by the circulation to a place which, like the preceeding, is only refusing anything and everything which presents, until it has at last found the love with which it can affiliate and the union occurs. This is secretion. How lovingly and eloquently we are taught that there should be bounds set by man, on his passions and appetites. Here we see no discords marring the harmony and symmetry of the existence of the elements. There is no voracious cannibalistic propensities. There we look in vain for divorce courts. These elements have found their affinities and death only comes when their mission is complete and perfect; when the transition occurs, so peacefully and quietly and imperceptibly that they have no funeral processions, no dirges, no requiums. There is also this peculiarity in regard to these affinities. We said in our last paragraph that the appropriating power was limited. Limited not only as to the affinity but also as to the amount. The cell, like an ounce bottle, can only take up the amount circumscribed by its capacity. This satisfied, the work ceases until the child is born and sent out on its mission, leaving behind it nothing but the space it occupied in its parents heart to be again possessed by new comers brought to its door by the blood. Now if the blood fails to thus bring with it this element as it enters the organ for which it has an affinity, the organ refuses to commit a fornication, or if you prefer an indiscretion and as a result its desires are not satisfied. It pines and withers and wrinkles, it refuses to be comforted and its career ends, when the effort to find that for which it has an affinity proves fruitless. This is death.

The importance then of directing our attention to the constituents of the blood, I mean the food we eat, is here made apparent. Unless we have the nutrient properties which satisfy the demands made by these different organs, the organs must cease to act and the result of such a cessation must be first, disease, then death. With the death of the organs, our hopes of building up tissue die also. When the little cells have performed their work and sent out the result of their affinities, we find the blood carrying hither and thither these products until they, like their grand parents, arrive at a threshold awaiting their coming.

The tissue is standing with eager straining eyes anxiously watching every passenger as he passes by the door until the well known face arrives. It has never seen it before, but it is nevertheless well known and it embraces it with an affectionate grasp until it spreads to its utmost limits in its new territory. From its start as a child from the cell, it has grown in its new relationship to be a mature being. It lengthens and broadens and deepens until it envelops within its folds, not only its parent cell but the house in which the parent cell lives, the organ and indeed is no niggard but refuses not to cover and protect several organs. Its usefulness is unlimited. It grasps with an iron hand, the femur and holds it to the tibia and fibula. It lays its affectionate arms around the inferior maxilla and brings it against its antagonist the superior. Thus by its kind offices, while it may call for its utmost powers of endurance, it assists in masticating the food which will at some future period be transformed into chyme and chyle, and enter the blood, which in its turn will bring straight to the scene of action occupied by it, perhaps to supplant it, perhaps to number its days of usefulness and drawing it from its abode, watch its decease with solicitude and care. Destiny requires that it stay not, it keeps pace with the demands made upon it, and dies like its predecessor with its work well done.

